# Global Coverage of Cetacean Line-Transect Surveys: Status Quo, Data Gaps and Future Challenges

**DOI:** 10.1371/journal.pone.0044075

**Published:** 2012-09-12

**Authors:** Kristin Kaschner, Nicola J. Quick, Rebecca Jewell, Rob Williams, Catriona M. Harris

**Affiliations:** 1 Evolutionary Biology and Ecology Lab, Institute for Biology I (Zoology), Albert-Ludwigs-University of Freiburg, Freiburg, Germany; 2 SMRU Ltd, New Technology Centre, St Andrews, Fife, United Kingdom; 3 Sea Mammal Research Unit, Scottish Oceans Institute, University of St Andrews, St Andrews, Fife, United Kingdom; 4 Centre for Research into Ecological and Environmental Modelling, University of St Andrews, St Andrews, Fife, United Kingdom; National Oceanic and Atmospheric Administration/National Marine Fisheries Service/Southwest Fisheries Science Center, United States of America

## Abstract

Knowledge of abundance, trends and distribution of cetacean populations is needed to inform marine conservation efforts, ecosystem models and spatial planning. We compiled a geo-spatial database of published data on cetacean abundance from dedicated visual line-transect surveys and encoded >1100 abundance estimates for 47 species from 430 surveys conducted worldwide from 1975–2005. Our subsequent analyses revealed large spatial, temporal and taxonomic variability and gaps in survey coverage. With the exception of Antarctic waters, survey coverage was biased toward the northern hemisphere, especially US and northern European waters. Overall, <25% of the world's ocean surface was surveyed and only 6% had been covered frequently enough (≥5 times) to allow trend estimation. Almost half the global survey effort, defined as total area (km^2^) covered by all survey study areas across time, was concentrated in the Eastern Tropical Pacific (ETP). Neither the number of surveys conducted nor the survey effort had increased in recent years. Across species, an average of 10% of a species' predicted range had been covered by at least one survey, but there was considerable variation among species. With the exception of three delphinid species, <1% of all species' ranges had been covered frequently enough for trend analysis. Sperm whales emerged from our analyses as a relatively data-rich species. This is a notoriously difficult species to survey visually, and we use this as an example to illustrate the challenges of using available data from line-transect surveys for the detection of trends or for spatial planning. We propose field and analytical methods to fill in data gaps to improve cetacean conservation efforts.

## Introduction

There are many compelling reasons to know how many cetaceans (whales, dolphins and porpoises) there are in a region, and how they are distributed in space and time. As top predators, cetaceans can exert strong influence on structuring marine ecosystems [1]. At the same time, cetacean populations may be impacted by a wide range of anthropogenic activities, including fishing operations [2], offshore wind farms [3], military sonar exercises [4] or accumulation of marine debris [5]. Many mitigating measures, such as those trying to ensure the sustainability of bycatch [6] or involving the site selection for noise-producing activities that minimize harmful exposure to acoustically sensitive cetaceans [4], [7] rely on good information on cetacean distribution and abundance in geographic areas of interest. Moreover, our ability to meet targets for halting biodiversity loss will hinge on our ability to first quantify local, regional and global species diversity. For various geographic regions, we therefore need to calculate biodiversity indices, which include number of species, overall abundance, and species evenness [8].

Enormous effort has gone into the investigation of cetacean abundance and distribution over the past 40 years. Despite these efforts, our current knowledge about many species remains very limited to the extent that the conservation status of a third of all currently recognized marine mammal species is unknown [9]. Similarly, the range extent of many species can only be derived from expert opinion [9] or from species distribution models that incorporate expert knowledge [10], [11]. In contrast, some geographic areas such as waters under US jurisdiction (and consequently the species occurring there), have been surveyed extensively. This extensive investment in cetacean population monitoring is driven by the US Marine Mammal Protection Act (MMPA), which provides quantitative guidance and places binding limits to the proportion of a marine mammal population that may be bycaught annually in commercial fisheries [6]. As part of the Census of Marine Life project, a global gap analysis was recently conducted to identify spatial and temporal gaps in occurrence data of higher marine vertebrates, compiled and available through the OBIS-SeaMap data repository [12]. However, a similar analysis to assess worldwide monitoring efforts used to estimate cetacean density and abundance and quantify existing gaps is still outstanding.

Such an overview of the current state of knowledge is required to provide a sound foundation for strategic planning of future survey efforts. Cetacean monitoring techniques include line-transect surveys, e.g. the SCANS surveys [13], [14], photo-identification studies [15], [16], [17] and counts of animals passing geographic points [18], [19] or newer methods estimating abundance of vocal species using passive acoustics [20], [21]. Among these techniques, visual line-transect surveys, conducted using ships or fixed-wing aircraft with dedicated marine mammal observers counting animals along pre-defined tracklines, are probably the most widely used method, particularly when trying to assess and quantify cetacean occurrence and density over large spatial scales [22], [23]. However, the large distribution and low detectability of many cetacean species requires large amounts of survey effort, to ensure sufficient sightings are made to estimate abundance. Combined with the vastness of the marine environment and the ecology of cetaceans, practical issues such as high expenditure and logistical challenges make it unlikely that there will ever be equal and frequent coverage of survey effort across all oceans. Therefore, given the limited resources available for conducting future surveys, taking stock of what has been achieved so far is the first step in a systematic conservation planning process [24] and also a pre-requisite to identify areas and species where survey coverage should be improved to ensure the most efficient usage of available funds and efforts.

Here, we provide an overview of the spatial, temporal and taxonomic coverage of the majority of existing visual line-transect surveys that report cetacean abundance using a geo-spatial database of marine mammal surveys conducted worldwide over 30 years until the year 2005. We identify and quantify gaps in global survey coverage in space and time and for different species. For a more in-depth analysis, we use the database to select an illustrative case study (the sperm whale, as it turns out) of a species whose range was surveyed with relatively high spatial and temporal coverage. For this comparatively data-rich species, we evaluate the extent to which existing survey coverage is sufficient to provide answers about temporal or spatial variation in species occurrence. Finally, we propose strategies to fill existing gaps through the optimization of future survey efforts and the development of models that may allow us to make inferences about species abundance in unsurveyed areas based on statistical relationships between empirical estimates of cetacean density and environmental conditions.

## Methods

Based on a comprehensive literature search including more than 1200 publications, we compiled data about the study sites, survey duration and reported species abundances of dedicated marine mammal surveys conducted around the world from the 1980s until 2005. We selected publications, including grey literature such as the US National Marine Fisheries Service (NMFS) reports [25], [26], [27], that have been reviewed by government or regulatory agencies. Taxonomically the focus lay on a pre-defined subset of 46 marine mammal species (including all ziphiids) that were of primary interest in the context of the Environmental Risk Management Capability (ERMC) project, which investigated how best to mitigate potential impacts of harmful anthropogenic activities such as intense acoustic disturbance [7]. However, information about other species was encoded if they were covered by the same surveys. We only included species-level estimates and did not use higher taxonomic level estimates often reported for those species which are typically difficult to distinguish at sea.

Within this database, comprehensive meta-data about each entered marine mammal survey are held, including the geographic location, the time period and duration, the size of the study area covered by the survey, survey platform and agency conducting the survey. Geographic attributes (e.g. study area, survey area) are defined below. In addition, abundance estimates and associated uncertainties were entered together with details relating to the abundance estimation (e.g. g(0)-correction of estimates, i.e. whether animals missed on the trackline have been accounted for) for all species for which this information was reported. Data were entered at the highest possible temporal and spatial resolution, i.e. where estimates for different strata or seasons were present within a survey, data were entered at this resolution. We encoded all data in a nested format so that each survey area could be linked to one or several survey blocks, and each survey block could be surveyed one or several times using the same or different methodology and be associated with one or several abundance estimates for different species. Therefore, from this point forward we use *survey* to refer to a discrete survey block for which an abundance estimate was produced for at least one species and at an individual time point. For example, each stratum within the SCANS I (Small Cetaceans Abundance in the North Sea and Adjacent Areas) project [13] is referred to here as a survey.

We geo-referenced all survey-related data by digitizing surveys in ArcGIS 9.1 to produce shapefiles of each survey study area, which were subsequently rasterized to re-express survey study areas geographically on the basis of complete or partial coverage of 0.5 degree latitude by 0.5 degree longitude cells. If cells were located along survey block boundaries, the area covered by the respective survey block falling within a boundary cell was calculated and the cell was included in the grids of all adjacent survey blocks. We also assigned each survey geographically to large scale ocean basins. In addition, we computed a number of latitudinal attributes of individual survey study areas, including the northern, southernmost and mid-latitudes based on the attributes of the associated half-degree grid cells.

In many cases, the actual size of a survey area was not reported in a paper and only a map or co-ordinates of the survey area were provided. The digitization process allowed us to calculate the size of each study area and allowed the conversion of abundance estimates into density estimates, thus making estimates directly comparable among surveys. To ensure consistency, we used calculated size to compute densities for all surveys, even when the area of a survey region was reported in the paper. The level of error associated with the survey area calculation was variable, due to different quality maps from the literature, differences in size of surveys and error associated with the digitization process. While the absolute error in the calculated area increased with survey size, the relative level of error was similar across surveys when quality of map was accounted for. The mean difference between reported and calculated area was 3.4% across all surveys for which the size of study site had been included in the publication.

We also assigned surveys and abundance estimates to specific time periods at different resolutions. First, we allocated surveys to the following seasonal categories: summer (surveys conducted during the months June–November in the northern hemisphere and during December to May in the southern hemisphere); non-summer (December–May in the northern and June–November in the southern hemisphere); and year-round (any survey covering longer than 6 months or spanning more than one season as defined above in either hemisphere). If abundance estimates were based on data collected over multiple years, we calculated the Mid-Year of the total data collection period.

To investigate the extent to which line-transect survey coverage and efforts might vary in time and space and for different species, we computed a number of survey related parameters for comparison. In this context we defined the *geographic survey area* as the total geographic size of the study area covered by a given survey, excluding all overlapping areas of temporally or spatially contiguous surveys. In contrast, we defined the *total survey effort* as the sum of the survey study area in terms of km^2^ covered by all surveys conducted over a specific time period. In both cases, we used the size of the study area as the basis for calculation, because of the frequent lack of information needed to obtain or calculate total track length and track line position in published sources. We assessed the extent of heterogeneity in survey effort (and geographic coverage) between different IUCN Marine Regions by comparing the observed survey effort in each region with the amount of effort that would be expected for each region based on its size alone if global survey effort had been distributed uniformly. In addition, to provide an estimate of how much of the known range of a species had been surveyed, we related geographic area covered by surveys to the predicted maximum range extent produced by a published model of relative environmental suitability (RES) model for each species [11].

To aid in visualization, we generated maps showing different aspects of the spatial and temporal global line-transect survey coverage. Frequency maps were produced by counting the number of times surveys with certain attributes were associated with a given half degree cell. The treatment of boundary grid cells between adjacent survey blocks described above resulted in multiple accounting of these cells, thus erroneously inflating survey coverage along such survey boundaries.

We used our database to select the species that has been covered by the most survey effort relative to its range. Using this example, (the sperm whale, *Physeter macrocephalus*), we illustrate further complexities in terms of seasonal and methodological variability of available cetacean abundance estimates that will affect the use of such data in population trend analysis or conservation planning exercises in detail.

## Results

### Spatial coverage and data gaps

**Figure 1:**
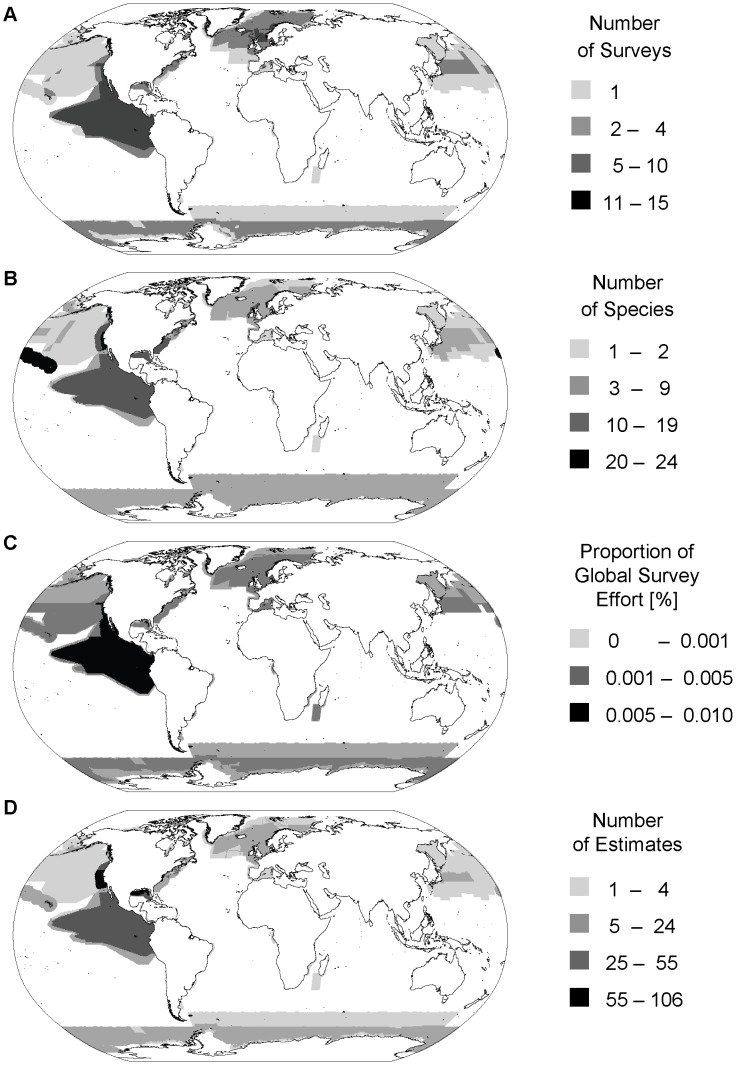
Global coverage of line-transect surveys by effort and species. (A) Survey effort in terms of frequency of coverage, (B) Percentage of global survey effort in terms of km^2^ falling into each cell, (C) Taxonomic coverage in terms of number of different species with reported abundance estimates, (D) Survey coverage in terms of number of reported abundance estimates. All data are shown as counts, or percent of global total respectively, per 0.5×0.5 degree grid cell. Note that darker lines within surveys represent survey block boundaries, where grid cells were counted multiple times, an artefact of the rasterization process.

**Figure 2:**
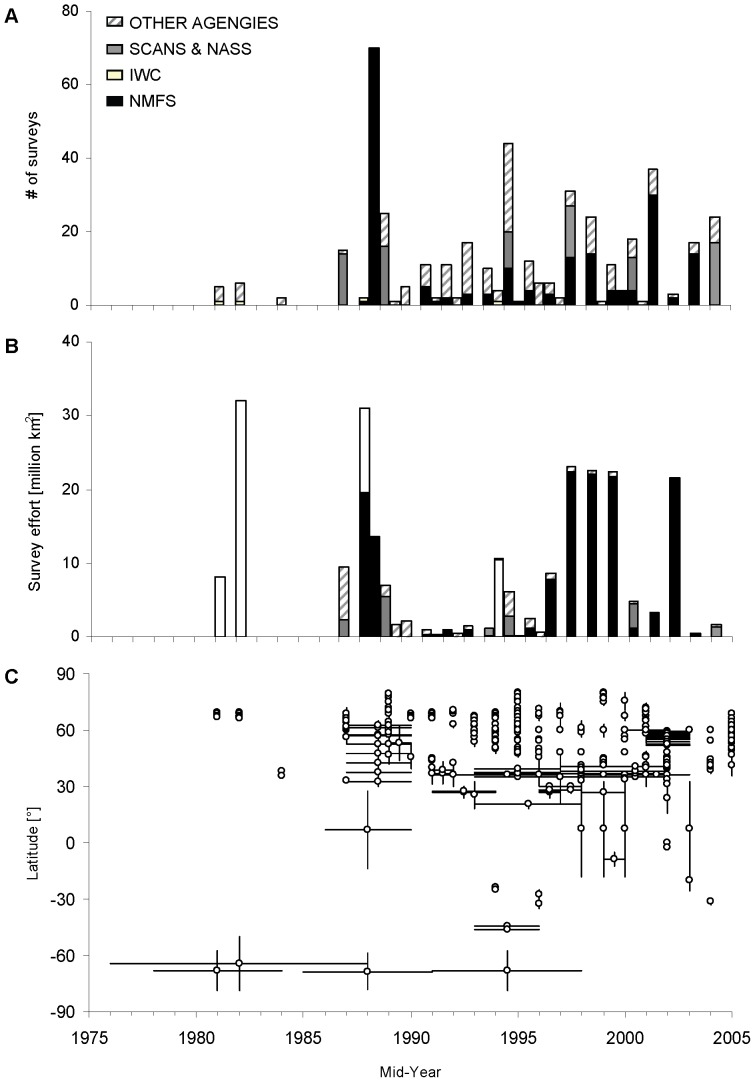
Spatial and temporal coverage of line-transect surveys. (A) Number of line-transect surveys conducted by mid-Year, (B) Total survey effort in km^2^ coverage by mid-year, (C) Latitudinal and temporal extent of survey coverage.

Our analysis included 430 surveys providing estimates for 47 cetacean species. Surveys were conducted by over 40 different research groups or agencies, covering over 320 different survey blocks from 31 geographically distinct locations over the course of our 30-year study period. However, of the ∼360 million km^2^ of the world's ocean surface, only ∼25% (i.e. ∼90 million km^2^) were covered by line-transect surveys during that time period. At the same time, total survey effort (as defined in the context of this study) amounted to more than 240 million km^2^. Geographic coverage was heterogeneously distributed and mostly concentrated in the northern hemisphere, particularly in waters under US and northern European jurisdiction (Fig. 1a). Proportional survey effort distribution was even more skewed (Fig. 1b), with almost half of the global line-transect effort (44%) being concentrated in the Eastern Tropical Pacific (ETP), an area corresponding to ∼6% of the ocean surface. Overall, two thirds of the world's global line-transect survey effort (66%) was carried out or led by US agencies over the study period (Fig. 2b). Heterogeneity of line-transect survey distribution was also apparent in a comparison of existing proportional survey coverage and effort per IUCN Marine Region versus the expected coverage based on the size of IUCN Marine Regions and an assumed uniform survey coverage (Table 1). The Baltic represented the only one of 18 IUCN Marine Regions where the actual survey coverage and effort corresponded to the expected coverage (Table 1). In contrast, in both Antarctic waters and the North East Pacific geographic survey coverage and effort were disproportionally high (3 times greater than expected; Table 1). In contrast, survey coverage in large parts of the Pacific and Indian Ocean as well as the South Atlantic was disproportionally low (Table 1).

**Table 1:** Distribution of survey coverage and effort by IUCN Marine Region.

IUCN Marine Region	Expected	Observed	
	Geographic Coverage & Effort [%]	Geographic Coverage [%]	Effort [%]
Antarctic	9.8	31.5	25.2
Arabian Sea	0.8	0.0	0.0
Arctic	6.4	10.1	9.3
Australia & New Zealand	9.4	4.0	1.6
Baltic	0.1	0.1	0.1
Central Indian Ocean	2.2	0.0	0.0
East Africa	6.1	0.7	0.3
East Asian Sea	3.4	0.0	0.0
Mediterranean & Black Sea	0.8	0.7	0.4
North East Atlantic	3.1	3.5	3.3
North East Pacific	6.3	22.3	23.3
North West Atlantic	1.9	1.0	0.8
North West Pacific	3.5	4.4	2.4
South Atlantic	4.3	0.9	0.4
South Pacific	24.5	10.4	13.9
South East Pacific	5.3	9.5	18.2
West Africa	8.9	0.1	0.0
Wider Caribbean	3.2	0.9	0.8

Observed percentage of total geographic survey coverage in km^2^ and effort (defined as sum of all km^2^ covered by line-transect surveys across all years of the study period), respectively, falling into each IUCN Marine Region. Expected survey effort was calculated based on the size of IUCN Marine Region and the assumption of homogeneous effort distribution.

### Temporal coverage and data gaps

Temporally, we assessed both inter- and intra-annual extent and patchiness of line-transect survey coverage. Geographic variation in survey frequency (i.e. coverage of an area by different surveys over time) varied as much as survey coverage itself (Fig. 1a). Frequently covered areas were mostly located in the Eastern Tropical Pacific, which had been surveyed regularly since the late 1980s (Fig. 2c) but also in the northern North Sea. Absolute frequency was highest in a small area at the mouth of Cook Inlet, Alaska, where the regularly conducted aerial surveys for beluga whales overlapped with Gulf of Alaska surveys (Fig. 1a). However, only a fifth of all surveyed areas were covered frequently enough by line-transect surveys to allow trend analysis of the data (assuming a minimum of 5 temporally distinct estimates per species per area are required for trend assessment). In total, these frequently surveyed areas represent only 6% of the world's ocean. In contrast, more than half of all surveyed areas, including most of the North Pacific as well as parts of the North Atlantic and Antarctic waters, were covered only once or twice (Fig. 1a).

Temporally, survey effort both with respect to number of surveys conducted and the area covered also greatly varied over the past 30 years. Any detection of trends in the number of surveys conducted per year (Fig. 2a) was greatly hampered by the varying levels of survey stratification over time, as reflected by the variation in latitudinal extent of surveys (Fig. 2c). Consequently, we found no relationship between the number of surveys conducted and the total area covered in each year (Fig. 2a & b). Conversely, the variation in overall survey effort in km^2^ each year was almost exclusively driven by a few very large surveys conducted in the ETP and Antarctic waters conducted by the US National Marine Fisheries Service [28], [29], [30], [31] and the International Whaling Commission (IWC) [32], [33], [34], [35] respectively (Fig. 2b & 2c). The overall contribution of these two agencies to the global survey effort was not directly apparent from the number of surveys conducted. However, US surveys represented the majority of all survey effort in more than half of the years during which any survey was conducted (Fig. 2a & 2b). Similarly, the contribution of the three Antarctic circumpolar IDCR/SOWER cruises (under the auspices of the IWC) to the overall effort is clearly apparent in Fig. 2b. Although masked by the large efforts of the US and the IWC, the North Atlantic Sightings Surveys (NASS) conducted in northern European waters (i.e. [36], [37], [38], [39] represented the third largest surveys in terms of effort (Fig. 2b).

In addition to inter-annual variation, survey coverage also showed extreme seasonal variation, with only 8.9% of surveys having been conducted exclusively during non-summer months, while the remaining survey effort consisted of summer or year-round surveys (Table 2). Although year-round surveys (dominated again by the very large tropical ETP surveys) only account for 20% of all surveys and 35% of the total geographic area, the associated total survey effort is greater than that of summer and non-summer effort combined (Table 2).

**Table 2:** Distribution of line transect survey coverage by season.

Season	Total Geographic Surveyed Area	Total Survey Effort	Number of Survey Areas Covered	Number of Surveys Conducted	Mean Surveyed Area
	[1000 km^2^]	[1000 km^2^]			[1000 km^2^]
NS	8,917	10,425	30	55	190
SU	60,062	100,241	214	289	347
YR	37,551	120,331	78	83	1,450

SU refers to surveys conducted in summer months in either hemisphere, NS to non-summer surveys and YR to year-round surveys.

### Taxonomic coverage and data gaps

The survey database contained 1120 species-level abundance estimates and associated uncertainty information for 47 cetacean species derived from visual line-transect surveys. Of these, 28 were ERMC focal species (highlighted in Table S1) for which the database coverage is fully comprehensive. For the remaining ERMC 18 focal species – mostly beaked whales and other species that are difficult to distinguish at sea – there were either no available species-level estimates or only estimates that were derived using monitoring techniques other than visual line-transect surveys. We estimate that, for ERMC focal species, we have incorporated ∼90% or more of all line-transect surveys conducted globally between 1975 and 2005 into the database, but some surveys of non-focal species may be missing from the data set. At the same time, although the database only contained data for roughly half of all known cetacean species, we estimate that these represent more than 85% of all cetaceans for which there are any available abundance estimates to be found in the literature, based on a comparison with cetacean surveys encoded in OBIS-Seamap and a review of available online literature reporting cetacean abundance estimates during our study period.

The number of reported abundance estimates available from line-transect surveys varied greatly geographically (Fig. 1d) as well as among species (Table S1). While regional abundance of some species had been estimated numerous times by large surveys, for other species information was much more sparse (Table S1). For instance, the database contains more than 100 abundance estimates for minke whales (*Balaenoptera acutorostrata* and *B. bonaerensis* which were combined in this analysis, because not all sources reliably distinguish between the two species), obtained from surveys covering a geographic area of ∼28 million km^2^ and twice that in terms of total survey effort (Table S1). In contrast, we only found one available species-level estimate for the Longman's beaked whale (*Indopacetus pacificus*), covering less than 2% of its predicted range (Table S1).

The proportion of each species' predicted geographical distribution that was surveyed was generally quite low (Table S1). For our 28 focal species, the average surveyed portion of each species distribution was only 12% (Table 1). The most notable exception in terms of proportional coverage was Dall's porpoise (*Phocoenoides dalli*). The high coverage (55%) for this species is an artifact of a relatively restricted distribution combined with one large, but non-systematic survey [40] in which observers on fisheries cruises collected opportunistic data on this species in the 1980s (Table S1 and Table S2). In contrast, for the beluga whale (*Delphinapterus leucas*), an area of <2% of the species' predicted range was surveyed, despite a high number of conducted surveys (Table S1). Except for three species, only a very small portion of each species distribution (<1%) had been surveyed frequently enough to allow any analysis of temporal trends (Table S1).

**Figure 3:**
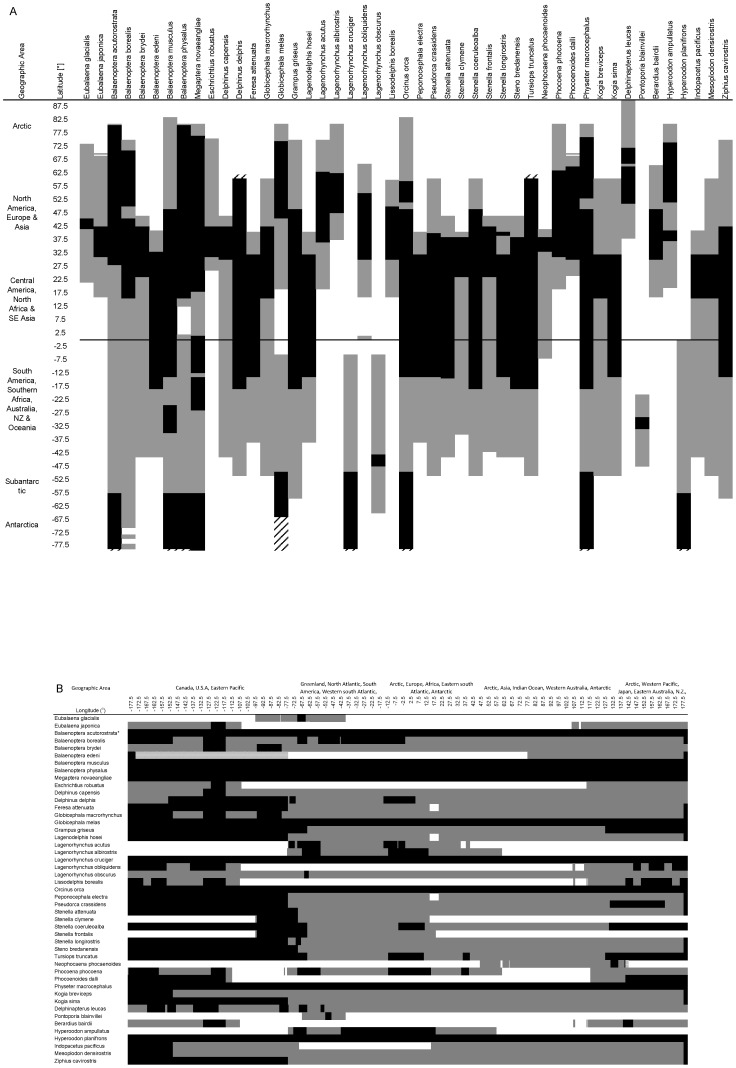
Comparison of survey coverage for each species with predicted distribution. (A) by latitude, (B) by longitude. Grey bands represent latitudinal or longitudinal extent of the species distribution as predicted by the RES model [11], black bands represent survey coverage of distribution and hatched areas represent areas covered by surveys for these species that go beyond boundaries of the predicted distribution. * includes *B. bonaerensis* surveys.

In terms of the latitudinal and longitudinal coverage of individual species distributions by surveys, our analysis showed that for most species, there had been little survey effort at the latitudinal margins of their distributional range (Figs. 3a & 3b). The contribution of the ETP surveys was again noticeable for many species occurring in the area (Figs. 3a & b). Latitudinal coverage of species ranges was better in the northern hemisphere than in the southern hemisphere, and for almost all species, there was a distinct lack of survey effort between 20°S and 50°S (Fig. 3a). Survey coverage was patchier in terms of longitude. The few species with seemingly comprehensive longitudinal survey coverage, such as *Balaenoptera* spp. and *Physeter macrocephalus* (Fig. 3b), are those covered by the IDCR/SOWER circumpolar surveys carried out in Antarctic waters (Fig. 1a). In the Pacific, for most species coverage was better in the eastern Pacific than in the western Pacific, especially when temporal aspects were taken into consideration (Figs. 3b & 1a). In contrast, there were large gaps in longitudinal coverage for many species throughout the Atlantic (Fig. 3b), and high survey effort e.g. the northeast Atlantic (Fig. 1a), mostly only translated to a small longitudinal coverage of species distribution (e.g. *Delphinus delphis* or *Lagenorhynchus acutus*) (Fig. 3b). When Figs. 3a and 3b are considered together, most species distributions were covered more extensively in terms of latitude than longitude, and line-transect survey coverage of most species was limited or missing throughout much of the South Pacific, South Atlantic and Indian Ocean. However, this picture would be even more exaggerated if the temporal dimension were included, since the shown latitudinal and longitudinal survey coverage of most species' distribution often corresponds to a single survey, which, in many cases, was conducted several decades ago (Fig. 1c & Figs. 3a & 3b).

### Multi-dimensional analysis of available line transect survey data: Sperm whale example

**Figure 4:**
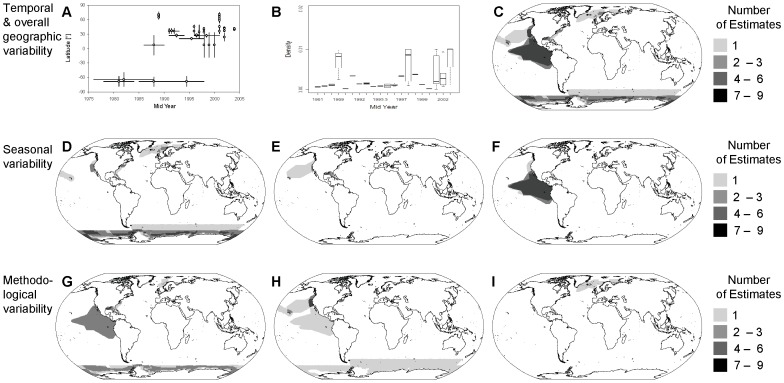
Data availability for large scale population trend analyses – sperm whale example. (A) Latitudinal and temporal coverage of available surveys of sperm whales, (B) reported sperm whale densities per mid-year. Maps show frequency of survey coverage of (C) all available data, (D) summer surveys, (E) non-summer surveys, (F) year-round surveys, (G) estimates from shipboard surveys not corrected for g(0), (H) estimates from shipboard surveys corrected for g(0) and (I) estimates from combined aerial and shipboard surveys not corrected for g(0).

We illustrate the extent of temporal, spatial and methodological variability within available surveys and abundance estimates that may affect any type of trend analysis or spatial planning exercise using one of the most spatially and temporally data-rich cetacean species to emerge from our analyses, namely the sperm whale (*Physeter macrocephalus*) (Fig. 4). The deep-diving behaviour of sperm whales makes this species notoriously difficult to survey using visual survey methods alone. Nevertheless, for this species, we had 49 available abundance estimates and the largest survey coverage both in terms of geographic area and overall effort (Table S1 & Fig. 4c). Despite this level of effort, only about 20% of the predicted distribution of this cosmopolitan species has been covered by surveys over the 30-year study period. Moreover, there are large temporal gaps in terms of consistent geographic survey coverage over the last three decades (Fig. 4a & c), leaving only a handful of geographic areas (such as the ETP, parts of the US west coast, the northern Gulf of Mexico and Antarctic waters) that were covered repeatedly over the course of several years, thus allowing the investigation of potential trends. Observed sperm whale density varied substantially not only between different years, but also intra-annually (Fig. 4b). At the same time, survey coverage varied greatly with seasons and in terms of methodological details such as e.g. consideration of animals missed on the trackline (Figs. 4d–i).

## Discussion

### Patterns emerging from the database: existing coverage, data gaps and associated caveats

Globally, a tremendous amount of effort has gone into surveying the distribution and abundance of cetaceans using visual shipboard and aerial line-transect surveys. Despite this effort, our analyses showed that substantial gaps remain: only a quarter of the world's ocean surface was covered by line-transect surveys over a 30-year time period and many areas were insufficiently or never surveyed. The geographic extent of line-transect surveys included in our analysis is relatively consistent with patterns shown by Kot et al. [12] based on OBIS-SeaMap (presence-only) data, which provides some support for the comprehensiveness of our analysis, although differences in taxonomic and temporal focus hinder direct comparison between the two studies.

Due to our pre-defined study period, which ended in 2005, our analysis did not include several large scale surveys conducted since then, most notably the Trans North Atlantic Sightings Survey (TNASS, http://www.nammco.no/Nammco/Mainpage/Tnass) led by the North Atlantic Marine Mammal Commission and the Cetacean Offshore Distribution and Abundance (CODA) [41] surveys in the European Atlantic offshore waters. However, TNASS and CODA study areas largely overlapped with previous NASS and SCANS surveys, and their inclusion would thus be unlikely to increase the overall geographic area covered by line-transect surveys by much (Fig. 1). Focusing only on visual line-transect surveys our maps obviously do not reflect all research efforts or represent the complete current state of knowledge about cetacean occurrence and abundance. A full representation of all available information in a common framework, however, will require the development of methods to convert other types of data, either from other monitoring methods or opportunistic sources into a common, area-independent currency (i.e. density).

The area covered by cumulative total km^2^ effort included in our database, is the equivalent of two-thirds of the global ocean surface, yet survey coverage was concentrated in only 25% of the world's ocean. This heterogeneity in effort distribution is probably best explained by national differences in existing legal requirements and measures implemented to protect marine mammals. The preponderance of surveys in waters under US jurisdiction, also clear from the analysis of trackline density by Kot et al. [12], makes sense given the objectives of the US MMPA, which among other parameters, requires reliable abundance estimates in national waters to prevent unsustainable bycatch of cetaceans and pinnipeds. The extreme concentration of survey effort in the ETP relative to the rest of the world (Fig. 1), for instance, is directly attributable to a long-standing bycatch issue of large numbers of pelagic dolphins in the purse-seine fisheries for yellow-fin tuna (*Thunnus albacares*) in this region [42]. Under the US MMPA, NMFS agencies are therefore required to assess sustainability of bycatch levels for depleted dolphin stocks by carrying out regular large-scale cruises covering the extensive area in which US fisheries operate [28], [43]. As a result, the survey coverage in the ETP gives us a benchmark of the quantity and quality of data needed to assess inter-annual (e.g., natural, climate-related and anthropogenic) variability in cetacean density in adequate detail [44], [45], [46], [47], [48], thus providing the basis for the development of adaptive management plans for conservation and human resource exploitation activities.

Although the map shown in Fig. 1a is probably a good representation of true survey effort, we will likely have over- or underestimated effort in some areas. Not all surveys that are conducted actually produce abundance estimates that are readily available through literature searches for reasons that include, among others, small sample size, lack of expertise or financial resources to carry out abundance estimation analyses, as well as the challenges of publishing papers in peer reviewed journals when the papers “only” report cetacean abundance. We searched for papers using search queries in English, which will have negatively biased the representation of some geographic regions, although the inclusion of work presented in national progress reports to the International Whaling Commission (the treaty organization with responsibility to manage cetacean populations) mitigates that bias to some degree. In addition, the nature of the cetacean survey literature and the myriad ways that researchers and regulatory agencies design, conduct and report results from cetacean line-transect surveys, often producing several estimates for the same species based on the same data pooled or analysed in different ways and across different time periods or areas complicates matters (compare e.g. [49]and [50] or [51], as reported in [52] or [53]). Effort in the ETP, as shown by our maps, for instance, represents an underestimate of true survey effort. Even though there were 12 survey cruises conducted in this area during our study period, earlier abundance estimates of our focal species occurring in the ETP are based on data pooled across several years [31]. Consequently, the associated study area was encoded as a single, multi-year survey thus effectively reducing the number of surveys represented in our database to five. Although there are some species for which estimates were available on a yearly basis, these were two of our non-focal species, the spotted dolphin, *Stenella attenuata*, and spinner dolphin, *S. longirostris*, [43], [46], for which data have not yet been comprehensively encoded. The inclusion of the missing surveys would, however, only further exaggerate the heterogeneity in survey effort distribution that is one of our main findings. Overestimation of existing survey effort is more difficult to pinpoint, but would be caused by duplicate inclusion of estimates based on the same data for the reasons described above. Despite all efforts to minimize duplication, a few errors will likely have remained in the database due to the complexity of the cetacean survey literature.

Direct inferences about patterns of global cetacean biodiversity based on our analysis and maps (Fig. 1c) are difficult for several reasons. The number of species with reported abundance estimates varies widely between different surveys, due to a combination of factors including differences in taxonomic focus and methodological limitations of individual surveys. For example, some surveys are designed specifically to estimate abundance for one species of interest [54] whilst others estimate abundance for as many species as possible within the region of interest [55]. Further adding to the complexity, the number of species detected during a survey has been shown to be directly proportional to survey effort [10] and in general, density estimation of rarely seen or cryptic species is difficult and therefore seldom attempted [33]. Nevertheless, new statistical estimation methods can salvage abundance estimates from surveys that yield smaller sample sizes than traditional rules of thumb [56], and the wealth of overall available data in some areas may make it possible to produce abundance estimates based on a single sighting [57]. Consequently, rare species that are often the cornerstone of conservation efforts, would mostly, but not consistently, be missing from Fig. 1c, thus further hampering their interpretation as the basis for biodiversity patterns.

At the opposite end of the spectrum, the spatial resolution of our analysis also affected the interpretation of latitudinal and longitudinal distribution of common species. The size of survey blocks created some artifacts and cases where surveyed areas actually exceeded the known range extent of species (e.g., abundance estimates of *Globicephala melas* in Fig. 3a were based on sightings from the most northern parts of IDCR/SOWER surveys, corresponding to the southern most extent of the species range. Nevertheless, in this analysis, estimates were subsequently linked to the entire survey extending down to the edge of the Antarctic continent [58]). Comparisons of patterns of taxonomic survey coverage (Fig. 1c) with global cetacean biodiversity patterns derived from expert maps or models [9], [10] thus show large discrepancies and highlight the problem of using survey data alone to compile regional species inventories.

### Future challenges: Detection of trends, conservation planning and filling the gaps

Our summary maps of visual line transect survey coverage are a first step towards a comprehensive quantitative assessment of existing knowledge of spatial and temporal variability in global cetacean occurrence and abundance. We take it as given that each individual survey in our database was sufficiently well designed, conducted and analyzed to allow the authors to meet their own objective. Political commitments to solve urgent global conservation issues, however, have created pressure to use existing data for a purpose for which they were not necessarily intended, namely to draw inference about large-scale cetacean distribution or trends over time. The future challenge therefore lies in synthesizing all these disparate monitoring efforts by developing methodologies for post-hoc meta-analyses [59], that may help to maximize the use of available data for addressing large-scale conservation questions.

The ability to detect changes in population sizes over time is a key aspect of any assessment of impacts of potentially harmful human activities on cetaceans. However, even in the best-studied regions (e.g., ETP, Gulf of Alaska, northern Gulf of Mexico or the US west coast) geographically and methodologically consistent surveys have rarely been replicated with sufficient frequency to allow detection of population trends before decreases in population sizes would exceed safe boundaries [60]. Our sperm whale case study illustrates just two of the many aspects of existing variation in survey coverage (Figs. 4d–i) that make it difficult to infer patterns or trends from Fig. 4b & 4c across or within years. As can be deduced from Figs. 4d–i, for this comparatively data-rich species, an assessment of inter-annual as well as intra-annual changes in species occurrence based on available line-transect data would have to be restricted to the US west coast – an area corresponding to only 0.25% of the species' predicted range. This is consistent with the patterns we found for other species, which for the most part had similarly small parts of their distribution where methodologically consistent survey coverage was sufficiently frequent for the reliable detection of trends (Table S1). Although new methods to assess cetacean population trends may require less data [61] and ocean-scale, cross-species meta-analyses can, in a few cases, be helpful [59], our findings stress the urgent need for strategic planning of methodologically and geographically consistent and frequent survey coverage in other areas.

The identification of target areas for protection hinges entirely on the analysis of patterns of species occurrence in space. The identification of such areas is one of the pre-requisites for the implementation of marine protected areas (MPAs) as part of the 2012 Marine Conservation Targets under the Convention on Biological Diversity [e.g. 62]. Systematic approaches to identify such areas based on scientific data [24], rely on spatial planning tools, such as Marxan [63] or C-Plan [64] that require all data to be in a common currency, and are highly sensitive to both patchiness and heterogeneity in input data [65]. This is a problem for cetacean conservation at a global scale, because much of the available information about cetacean occurrence exists in the form of opportunistic sightings from which no inferences can be drawn about the relative importance of different areas for different species due to lack of associated effort information. Other more quantitative investigations of absolute species occurrence based on photo-identification studies or passive acoustic monitoring are difficult to allocate to specific areas, thus hindering their use in marine spatial planning exercises. However, our exercise has shown that even data from visual line-transect surveys that are apparently available in a common currency (i.e. density) in reality represent ‘apples and oranges’. As shown by our sperm whale example, available density estimates cannot be assumed to be directly comparable across studies and agencies due to differences in survey methodology, data analyses and intra-annual and inter-annual temporal coverage. For instance, only a third of all cetacean density estimates encoded in our database had accounted for animals missed on the trackline (i.e., g(0)<1). Because of this disparity, uncorrected, (i.e., negatively biased) density estimates will cause survey regions to appear less “important” to marine spatial planning algorithms than those that do account for g(0)<1, when really, these apparent differences just reflect differences in survey methodology.

Similarly, the extreme heterogeneity of survey coverage and effort shown in our analysis, would heavily bias any results from spatial planning algorithms toward ETP and other data-rich areas [65], [66]. At the same time, the direct relationship between the number of species sighted in an area and survey effort and the incompleteness of regional species inventories based on surveys alone [10] represent further confounding factors. As a result, outputs from spatial planning exercises may further gravitate to data-rich areas where a greater proportion of species diversity present will have been detected than in more sparsely surveyed regions [66]. Finally, however, the extreme patchiness of line-transect survey coverage itself (Fig. 3 & Fig. 4) probably represents the greatest barrier for the direct use of available data sets in spatial planning algorithms, because most algorithms require the distinction between true species absences from absence of effort [63], [65]. The data layer summarizing frequency of survey coverage compiled during this analysis and made available for download (Table S3) may help to account for global variability in line-transect survey effort in future marine spatial planning exercises.

Hence, despite the quantity and good quality of available cetacean densities from visual line-transect surveys, existing data at the moment can only be considered to represent an incomplete snapshot of cetacean distribution at a global scale. Although we fully acknowledge the considerable logistical and technical challenges to fill existing gaps, additional efforts in development of both field and analytical methods are urgently needed to produce an unbiased estimate, however coarse, of the average distribution of cetacean species across the world's ocean. Ideally, analytical and field methods would be employed in an iterative fashion, in which models are constructed from existing data to predict density in a random or systematic sample of unsurveyed waters; predictions are then field-tested with newly collected data and the models updated accordingly.

In general, it is preferable to base management decisions on empirical data rather than model predictions. However, given the high logistical and financial costs of dedicated line-transect surveys, the quest for representative coverage of the marine environment at regular intervals is a highly unrealistic option. Small-boat surveys and data from platforms of opportunity or non-randomized surveys [67] may represent cost-efficient ways to fill some gaps, but both methods come with their own sets of constraints in terms of geographic coverage. Similarly, emerging passive acoustic monitoring methods may supplement visual line-transect coverage, but these will remain to be restricted to vocal species [20]. As a priority though, future survey effort should ideally be conducted strategically in a coordinated way through international collaborations, building upon existing large-scale efforts such as the IDCR/SOWER, TNASS and CODA surveys to fill existing gaps. Thomas et al. [68] outline a method for dividing a survey region into substrata that can be randomly selected to keep the cost of field surveys low. It is hoped that our maps will serve as a starting point for the development of a cost-effective global strategy based on a survey design that uses randomized subsets of unsurveyed areas to maximize our ability to make inferences about cetacean distribution at a global scale.

Even under the best circumstances, coverage of cetacean distribution through line-transect surveys will remain patchy in the foreseeable future. An emphasis should therefore be put on developing methods to standardize outputs from different monitoring or analyses techniques. This could, for example, include meta-analyses of the data compiled in our database to account for detectable survey biases (e.g. animals missed on the trackline or responsive movement) to make line-transect based densities estimates more directly comparable [59]. In addition, statistical models that predict species occurrence or density surfaces from line transect survey data [69] based on local environmental conditions could be expanded to make inferences about cetacean densities in unsurveyed areas. Whitehead [70], for instance, estimated global sperm whale abundances by extrapolating the statistical relationship between observed densities and environmental conditions in survey areas to unsurveyed areas, based on the assumption that combined survey effort for this species spanned the global range of model covariates. Similarly, but replacing environmental conditions with outputs from a large-scale species distribution model [11], Kaschner et al. (unpublished data) produced preliminary global density estimates of 46 marine mammal species from spatially and temporally nested regression models as part of the ERMC project [7]. Although extensive validation is still outstanding and uncertainties associated with density predictions are necessarily very high, these predicted surfaces, at the very least, should give a way of making interim inferences about how much difference it would make to critical habitat predictions or MPA network designs if data from currently unsurveyed areas were to become available.

In summary, despite tremendous effort, coverage of ocean surfaces by cetacean line-transect surveys was highly variable with extreme concentrations of effort in relatively small areas. To maximize use of available data and future resources to address pressing conservation issues, we propose the development of a comprehensive global strategy for cetacean monitoring efforts including the advancement of models that allow inferences to be made about cetacean occurrence and densities in unsurveyed areas. Our assessment of existing data has identified a number of caveats that would need to be considered, but the database also provides a comprehensive foundation on which subsequent analyses can be based that would inform a range of management objectives and address the needs of conservation planners.

## Supporting Information

Table S1 **Analysis of line transect survey coverage by species.** List of species covered in database and number of encoded abundance estimates. Also shown is the proportion of estimates corrected for g(0), the total geographic area surveyed and the resulting percentage of the known distribution covered by any line-transect surveys as well as frequently surveyed (≥5 times) portions and total survey effort in km^2^ over time. Focal cetacean species covered by line-transect surveys with available species level estimates (n = 28) are highlighted in bold. For these species encoded data probably represents a comprehensive coverage of existing surveys conducted during the study period. Note that errors associated with the digitization and rasterization process are responsible for some erroneous discrepancies between total survey effort and geographic survey area. * includes *B. bonaerensis* surveys.
(DOC)

Table S2 **List of surveys included in the analysis.** Includes information on name and nationality of lead survey agency or institution (defined as affiliation of main author listed in source), geographic location code (N = North, S = South, E = East, W = West, A = Atlantic, P = Pacific, M = Mediterranean, I = Indian Ocean, R = Arctic, T = Antarctic, U = ubiquitous, i.e. longitudinal or latitudinal coverage spanning several hemispheres or entire ocean basins), description of geographic survey area, the year the area was first and last covered by surveys and the maximum number of times any part of the survey area was covered as well as the maximum number of sub-survey areas covered at any point in time and the published source from which the information was extracted. Please note that more detailed information about specific geographic areas, species covered, estimated abundance and densities, survey types and methodologies are available upon request from the first author at Kristin.kaschner@biologie.uni-freiburg.de.
(DOC)

Table S3 **Data layer for frequency of survey coverage per 0.5 degree grid cell.** Contains the following columns: CSQ – unique cell id of each 0.5 degree cell based on c-squares; Lat & Lon – center latitude/longitude of each 0.5 degree grid cell, Number of Surveys = total number of surveys, as defined in the context of this analysis, covering this grid cell.
(ZIP)
