# Supplementary material for: Global Coverage of Cetacean Line-Transect Surveys: Status Quo, Data Gaps and Future Challenges
Source: PLoS One. 2012 Sep 12;7(9):e44075. doi: 10.1371/journal.pone.0044075 (PMC3440399; doi:10.1371/journal.pone.0044075)
Supplement: Table S1 — Analysis of line transect survey coverage by species. List of species covered in database and number of encoded abundance estimates. Also shown is the proportion of estimates corrected for g(0), the total geographic area surveyed and the resulting percentage of the known distribution covered by any line-transect surveys as well as frequently surveyed (≥5 times) portions and total survey effort in km2 over time. Focal cetacean species covered by line-transect surveys with available species level estimates (n = 28) are highlighted in bold. For these species encoded data probably represents a comprehensive coverage of existing surveys conducted during the study period. Note that errors associated with the digitization and rasterization process are responsible for some erroneous discrepancies between total survey effort and geographic survey area. * includes B. bonaerensis surveys. (DOC) [file pone.0044075.s001.doc]

**Supplementary Materials Table S1: Analysis of line-transect survey coverage by species.**

| Family | Scientific name | Number of abundance estimates | G(0)-corrected estimates | Total geographic surveyed area | Portion of distribution surveyed | Portion of distribution surveyed > 5 times | Survey effort |
| --- | --- | --- | --- | --- | --- | --- | --- |
|  |  |  | [%] | [1000 km^2^] | [%] | [%] | [1000 km^2^] |
| Balaenidae | *Eubalaena glacialis* | 5 | 100.0 | 93 | 1.0 | 0.0 | 119 |
| Balaenidae | *Eubalaena japonica* | 1 | 100.0 | 264 | 0.7 | 0.0 | 261 |
| **Balaenopteridae** | ***Balaenoptera acutorostrata**** | **119** | **20.2** | **27,705** | **8.0** | **0.1** | **51,336** |
| **Balaenopteridae** | ***Balaenoptera borealis*** | **28** | **35.7** | **6,085** | **1.9** | **0.0** | **8,483** |
| Balaenopteridae | *Balaenoptera brydei* | 5 | 20.0 | 1,263 | 0.5 | 0.0 | 1,849 |
| Balaenopteridae | *Balaenoptera edeni* | 6 | 16.7 | 25,102 | 34.9 | 0.0 | 86,830 |
| Balaenopteridae | *Balaenoptera musculus* | 15 | 60.0 | 38,251 | 10.9 | 0.1 | 55,453 |
| **Balaenopteridae** | ***Balaenoptera physalus*** | **93** | **32.3** | **27,170** | **12.6** | **0.0** | **45,410** |
| Balaenopteridae | *Megaptera novaeangliae* | 90 | 30.0 | 24,667 | 7.1 | 0.1 | 43,979 |
| Eschrichtiidae | *Eschrichtius robustus* | 1 | 100.0 | 264 | 2.8 | 0.0 | 261 |
| Delphinidae | *Delphinus capensis* | 3 | 100.0 | 834 | 0.4 | 0.0 | 2,492 |
| **Delphinidae** | ***Delphinus delphis*** | **33** | **36.4** | **24,201** | **8.9** | **6.4** | **101,736** |
| **Delphinidae** | ***Feresa attenuata*** | **6** | **16.7** | **22,388** | **11.3** | **0.0** | **23,301** |
| **Delphinidae** | ***Globicephala macrorhynchus*** | **9** | **33.3** | **3,750** | **1.4** | **0.0** | **5,672** |
| **Delphinidae** | ***Globicephala melas*** | **34** | **2.9** | **36,782** | **33.6** | **0.0** | **40,265** |
| **Delphinidae** | ***Grampus griseus*** | **33** | **63.6** | **31,265** | **10.5** | **0.1** | **54,007** |
| Delphinidae | *Lagenodelphis hosei* | 6 | 16.7 | 22,395 | 11.3 | 0.0 | 22,964 |
| **Delphinidae** | ***Lagenorhynchus acutus*** | **21** | **76.2** | **682** | **5.1** | **0.0** | **978** |
| **Delphinidae** | ***Lagenorhynchus albirostris*** | **19** | **21.1** | **1,241** | **10.0** | **0.0** | **1,807** |
| Delphinidae | *Lagenorhynchus cruciger* | 1 | 0.0 | 31,720 | 30.0 | 0.0 | 31,979 |
| **Delphinidae** | ***Lagenorhynchus obliquidens*** | **33** | **18.2** | **6,166** | **16.6** | **0.0** | **8,630** |
| Delphinidae | *Lagenorhynchus obscurus* | 2 | 0.0 | 45 | 0.1 | 0.0 | 45 |
| **Delphinidae** | ***Lissodelphis borealis*** | **20** | **30.0** | **4,149** | **12.5** | **0.0** | **6,489** |
| **Delphinidae** | ***Orcinus orca*** | **33** | **27.3** | **57,495** | **16.2** | **0.1** | **89,670** |
| **Delphinidae** | ***Peponocephala electra*** | **8** | **12.5** | **22,389** | **11.3** | **0.0** | **23,528** |
| Delphinidae | *Pseudorca crassidens* | 9 | 11.1 | 25,596 | 9.5 | 0.0 | 26,654 |
| Delphinidae | *Stenella attenuata* | 25 | 4.0 | 13,735 | 5.7 | 4.4 | 55,928 |
| Delphinidae | *Stenella clymene* | 6 | 0.0 | 1,014 | 2.1 | 0.0 | 2,022 |
| **Delphinidae** | ***Stenella coeruleoalba*** | **31** | **41.9** | **32,139** | **11.8** | **0.0** | **118,581** |
| **Delphinidae** | ***Stenella frontalis*** | **17** | **23.5** | **1,207** | **2.1** | **0.0** | **2,716** |
| Delphinidae | *Stenella longirostris* | 18 | 11.1 | 12,370 | 5.2 | 4.1 | 60,233 |
| Delphinidae | *Steno bredanensis* | 10 | 10.0 | 25,769 | 10.6 | 0.0 | 45,519 |
| **Delphinidae** | ***Tursiops truncatus*** | **64** | **40.6** | **32,708** | **12.1** | **0.1** | **55,593** |
| **Phocoenidae** | ***Neophocaena phocaenoides*** | **4** | **0.0** | **22** | **0.4** | **0.0** | **22** |
| **Phocoenidae** | ***Phocoena phocoena*** | **84** | **60.7** | **2,536** | **18.9** | **0.0** | **4,351** |
| **Phocoenidae** | ***Phocoenoides dalli*** | **84** | **7.1** | **13,484** | **55.2** | **0.1** | **16,373** |
| **Physeteridae** | ***Physeter macrocephalus*** | **49** | **46.9** | **70,760** | **20.3** | **0.1** | **167,136** |
| Kogiidae | *Kogia breviceps* | 1 | 100.0 | 2,472 | 0.9 | 0.0 | 2,477 |
| Kogiidae | *Kogia sima* | 2 | 50.0 | 21,946 | 8.2 | 0.0 | 21,995 |
| **Monodontidae** | ***Delphinapterus leucas*** | **57** | **45.6** | **423** | **1.4** | **0.4** | **1,043** |
| Pontoporiidae | *Pontoporia blainvillei* | 1 | 100.0 | 15 | 1.8 | 0.0 | 15 |
| **Ziphiidae** | ***Berardius bairdii*** | **7** | **71.4** | **1,297** | **3.5** | **0.0** | **2,499** |
| **Ziphiidae** | ***Hyperoodon ampullatus*** | **11** | **9.1** | **3,686** | **21.4** | **0.0** | **5,281** |
| **Ziphiidae** | ***Hyperoodon planifrons*** | **2** | **0.0** | **15,582** | **10.6** | **0.0** | **22,131** |
| **Ziphiidae** | ***Indopacetus pacificus*** | **1** | **100.0** | **2,472** | **1.4** | **0.0** | **2,477** |
| **Ziphiidae** | ***Mesoplodon densirostris*** | **2** | **50.0** | **2,473** | **0.9** | **0.0** | **2,562** |
| **Ziphiidae** | ***Ziphius cavirostris*** | **11** | **36.4** | **23,224** | **7.7** | **0.0** | **26,020** |
