# Supplementary material for: Global Coverage of Cetacean Line-Transect Surveys: Status Quo, Data Gaps and Future Challenges
Source: PLoS One. 2012 Sep 12;7(9):e44075. doi: 10.1371/journal.pone.0044075 (PMC3440399; doi:10.1371/journal.pone.0044075)
Supplement: Table S2 — List of surveys included in the analysis. Includes information on name and nationality of lead survey agency or institution (defined as affiliation of main author listed in source), geographic location code (N = North, S = South, E = East, W = West, A = Atlantic, P = Pacific, M = Mediterranean, I = Indian Ocean, R = Arctic, T = Antarctic, U = ubiquitous, i.e. longitudinal or latitudinal coverage spanning several hemispheres or entire ocean basins), description of geographic survey area, the year the area was first and last covered by surveys and the maximum number of times any part of the survey area was covered as well as the maximum number of sub-survey areas covered at any point in time and the published source from which the information was extracted. Please note that more detailed information about specific geographic areas, species covered, estimated abundance and densities, survey types and methodologies are available upon request from the first author at Kristin.kaschner@biologie.uni-freiburg.de. (DOC) [file pone.0044075.s002.doc]

**Supplementary Materials Table S2.**

| **Survey Agency** | **Country of Survey Agency** | **Survey Location** | **Geographic Area Covered by Survey** | **First Surveyed** | **Last Surveyed** | **Max. Number of Times Surveyed** | **Max. Number of Survey Areas Covered** | **Source** |
| --- | --- | --- | --- | --- | --- | --- | --- | --- |
| Greenland Institute of Natural Resources | Greenland/ Denmark | NEA | Baffin Bay & Greenland Sea | 2005 | 2005 | 1 | 5 | Heide-Jørgensen et al. 2006 |
| North Atlantic Sightings Survey | International Body | NEA | Barents Sea, Bay of Biscay, Faroer Islands, Greenland Sea, Iceland Sea, North Sea, Norwegian Sea (NASS-87, NASS-89, NASS-95 & NASS-01) | 1987 | 2001 | 4 | 23 | Buckland & Cattanach 1992, Buckland et al. 1993a, Cattanach et al. 1993, Christensen et al. 1992, C. Paxton, pers comm, Gunnlaugsson & Sigurjónsson, 1990 |
| Institute of Marine Research | Norway | NEA | Barents Sea, Greenland Sea, Iceland Sea, North Sea, Norwegian Sea | 1989 | 2001 | 4 | 19 | Christensen et al. 1992, Schweder et al. 1997, Skaug et al. 2004, |
| Coastal & Marine Research Centre, University College Cork | Ireland | NEA | Celtic Sea | 2000 | 2000 | 1 | 1 | O'Cadhla et al. 2001 |
| Forschungs- und Technologie Zentrum Westkueste | Germany | NEA | North Sea & Baltic | 1995 | 1996 | 2 | 3 | Siebert et al. 2006 |
| Forschungs- und Technologie Zentrum Westkueste | Germany | NEA | North Sea & Baltic | 2004 | 2005 | 2 | 2 | Scheidat et al. 2006 |
| Sea Mammal Research Unit | UK | NEA | North Sea and adjacent waters (SCANS I & II) | 1994 | 2005 | 2 | 17 | Hammond et al. 2002, Burt et al. 2006 |
| Sea Mammal Research Unit | UK | NEA | West coast of Scotland | 1998 | 1998 | 1 | 2 | Macleod 2004, MacLeod et al. 2006 |
| ALNITAK Marine Research Center | Spain | NWM | Mediterranean Sea | 1992 | 2003 | 3 | 2 | Canadas & Hammond 2006 |
| British Antarctic Surveys | Spain | NWM | Mediterranean Sea | 2001 | 2002 | 2 | 3 | Forcada et al. 2004 |
| Department of Animal Biology, University of Barcelona | Spain | NWM | Mediterranean Sea | 1991 | 1991 | 1 | 1 | Forcada et al. 1994 |
| Tethys Research Institute | Italy | NWM | Mediterranean Sea | 1992 | 1992 | 1 | 1 | Forcada et al. 1995 |
| BREMA Laboratory | Ukraine | NEM | Black Sea | 2001 | 2002 | 2 | 4 | Birkun et al. 2003 |
| National Marine Fisheries Service | USA | NEP | Hawaii | 2002 | 2002 | 1 | 1 | Barlow 2003b, Barlow, 2006 |
| National Marine Fisheries Service | USA | NEP | Hawaii | 1993 | 1998 | 1 | 1 | Mobley et al. 2000 |
| National Marine Fisheries Service | USA | NEP | Temperate NE Pacific | 1997 | 2000 | 2 | 1 | Barlow & Taylor, 2005 |
| Cascadia Research | USA | NEP | US West Coast | 1995 | 2002 | 5 | 2 | Calambokidis et al. 2004 |
| Cascadia Research | USA | NEP | US West Coast | 1991 | 1996 | 2 | 4 | Calambokidis & Barlow 2004, Forney et al. 1995 |
| National Marine Fisheries Service | USA | NEP | US West Coast | 1993 | 1997 | 2 | 4 | Forney 1999, Carretta et al. 2001 |
| National Marine Fisheries Service | USA | NEP | US West Coast | 1991 | 1991 | 1 | 1 | Forney & Barlow, 1993 |
| National Marine Fisheries Service | USA | NEP | US West Coast | 1997 | 2002 | 2 | 4 | Carretta 2003, Carretta & Forney 2004 |
| National Marine Fisheries Service | USA | NEP | US West Coast | 1991 | 2001 | 3 | 2 | Barlow, 2003a |
| National Marine Fisheries Service | USA | UEP | Eastern Tropical Pacific (ETP) | 1986 | 2003 | 5 | 9 | Wade & Gerrodette 1993, Gerrodette & Forcada 2002a, b, Gerrodette et al. 2005 |
| National Marine Fisheries Service | USA | NUP | Temperate N Pacific | 1987 | 1990 | 1 | 70 | Buckland et al. 1993b |
| National Marine Fisheries Service | USA | NEP | Gulf of Alaska | 2001 | 2003 | 1 | 16 | Zerbini et al. 2006, Zerbini et al. 2007 |
| National Marine Fisheries Service | USA | NEP | Gulf of Alaska | 1994 | 2004 | 11 | 1 | Hobbs et al. 2000, Rugh et al. 2005 |
| Alaska Department of Fish and Game | USA | NUP | Alaska | 1992 | 1995 | 5 | 2 | Lowry et al. 1999a, b |
| National Marine Fisheries Service | USA | NUP | Bering Sea | 1999 | 2000 | 1 | 2 | Moore et al. 2000, Moore et al 2002, Waite et al. 2002 |
| Department of Fisheries and Oceans | Canada | NUR | Beaufort Sea | 1992 | 1992 | 1 | 8 | Harwood et al. 1996 |
| Greenland Fisheries Research Institute | Greenland/ Denmark | NWA | Baffin Bay | 1993 | 1993 | 1 | 6 | Larsen 1995 |
| Greenland Fisheries Research Institute | Greenland/ Denmark | NWA | Baffin Bay | 1981 | 1994 | 6 | 5 | Heide-Jørgensen et al. 1993, Heide-Jørgensen & Reeves, 1996 |
| Department of Fisheries and Oceans | Canada | NUR | Canada East Coast | 1993 | 1993 | 2 | 4 | Kingsley 2000 |
| Department of Fisheries and Oceans | Canada | NWA | Canada East Coast | 1995 | 1996 | 1 | 4 | Kingsley & Reeves 1998 |
| National Marine Fisheries Service | USA | NWA | US East Coast | 1998 | 2004 | 3 | 13 | Palka 2006 |
| National Marine Fisheries Service | USA | NWA | US East Coast | 2002 | 2002 | 1 | 3 | Garrison et al. 2003 |
| National Marine Fisheries Service | USA | NWA | US East Coast | 1999 | 1999 | 1 | 4 | Clapham et al. 2003 |
| National Marine Fisheries Service | USA | NWA | US East Coast | 1998 | 1998 | 1 | 1 | Mullin & Fulling 2003 |
| National Marine Fisheries Service | USA | NWA | US East Coast | 1991 | 1991 | 1 | 4 | Palka 1995 |
| Ecology of Harmful Algal Blooms, University of South Florida | USA | NWA | Gulf of Mexico | 1998 | 2000 | 1 | 1 | Griffin & Griffin 2002 |
| National Marine Fisheries Service | USA | NWA | Gulf of Mexico | 1991 | 2001 | 3 | 3 | Blaylock et al. 1995, Hansen et al. 1995, Davis & Fargion 1996, Davis et al. 2000, Mullin & Fulling 2004 |
| Japan Fisheries Agency | Japan | NWP | Japan | 1984 | 1984 | 1 | 2 | Miyashita 1986 |
| National Research Institute of Far Seas Fisheries | Japan | NWP | Japan | 1983 | 1991 | 1 | 5 | Miyashita 1993 |
| National Research Institute of Far Seas Fisheries | Japan | NWP | Japan | 1991 | 1992 | 1 | 1 | Miyashita & Kato 1993 |
| Ocean Research Institute, University of Tokyo | Japan | NWP | Japan | 2000 | 2000 | 1 | 3 | Shirakihara et al. 2007 |
| Ocean Research Institute, University of Tokyo | Japan | NWP | Japan | 2000 | 2000 | 1 | 1 | Amano et al. 2003 |
| National Research Institute of Far Seas Fisheries | Japan | NWP | Japan & Sea of Okhotsk | 1989 | 1990 | 1 | 2 | Buckland et al. 1992 |
| Centro Nacional Patagonico and Centro Austral de Investigaciones Cientificas | Argentina | SWA | Argentina | 1993 | 1996 | 1 | 2 | Schiavini et al. 1999 |
| Grupo de Estudos de Mamıferos Aquáticos do Rio Grande do Sul | Brazil | SWA | Brazil | 2004 | 2004 | 1 | 1 | Danilewicz et al. 2006 |
| University of Washington | Brazil | SWA | Brazil | 1999 | 2000 | 1 | 1 | Zerbini et al. 2004 |
| Wildlife Conservation Society | USA | SEA | Gabon | 2002 | 2002 | 1 | 2 | Rosenbaum et al. 2004 |
| International Whaling Commission | International Body | SWI | Madagaskar | 1994 | 1996 | 2 | 2 | Best et al. 1996, Best et al. 2003 |
| University of Cape Town | South Africa | SWI | Mozambique | 2003 | 2003 | 1 | 1 | Rosenbaum et al. 2004 |
| International Whaling Commission | International Body | SUT | Antarctic | 1978 | 1998 | 1 | 3 | Branch & Butterworth 2001a, b |
| International Whaling Commission | Japan | SUT | Antarctic | 1976 | 1988 | 1 | 1 | Kasamatsu & Joyce 1995 |

**References**

Amano A, Nakahara F, Hayano A, Shirakihara K (2003) Abundance estimate of finless porpoises off the Pacific coast of eastern Japan based on aerial surveys. Mammal Study 28:103-110

Barlow J (2003a) Cetacean abundance in Hawaiian waters during summer/fall 2002. Report No. Admin. Rept. LJ-03-13, Southwest Fisheries Science Center (SWFSC), National Marine Fisheries Service (NMFS), National Oceanic and Atmospheric Administration (NOAA), La Jolla, CA, USA

Barlow J (2003b) Preliminary estimates of the abundance of cetaceans along the U.S. west coast: 1991-2001. Report No. Admin. Rept. LJ-03-03, Southwest Fisheries Science Center (SWFSC), National Marine Fisheries Service, (NMFS), National Oceanic and Atmospheric Administration (NOAA), La Jolla, CA, USA

Barlow J (2006) Cetacean abundance in Hawaiian waters estimated from a summer/fall survey in 2002. Marine Mammal Science 22:446-464

Barlow J, Taylor BL (2005) Estimates of sperm whale abundance in the northeastern temperate Pacific from a combined acoustic and visual survey. Marine Mammal Science 21:429-445

Best PB (2003) The abundance of blue whales on the Madagascar Plateau, December 1996. Journal of Cetacean Research and Management 5:253–260

Best PB, Sekiguchi K, Rakotonirina B, Rossouw A (1996) The distribution and abundance of humpback whales off southern Madagascar, August-September 1994. Reports of the International Whaling Commission 46:323

Birkun A, Glazov D, Krivokhizhin S, Mukhametov L (2003) Distribution and abundance estimates of cetaceans in the Azov Sea, Kerch Strait and northeastern shelf area of the Black

Sea: Results of aerial surveys in July 2001 and August 2002 (SC/55/SM15) International Whaling Commission - Scientific Committee Meeting. (unpublished), Berlin, Germany, p 8

Blaylock RA, Hain JHW, Hansen LJ, Palka DL, Waring GT (1995) U.S. Atlantic and Gulf of Mexico stock assessments. Report No. NMFS-SEFSC-363, U.S. Department of Commerce

Branch TA, Butterworth DS (2001a) Estimates of abundance south of 60 degree S for cetacean species sighted frequently on the 1978/79 to 1997/98 IWC/IDCR-SOWER sighting surveys. Journal of Cetacean Research and Management 3:251-270

Branch TA, Butterworth DS (2001b) Southern hemisphere minke whales: Standardized abundance estimates from the 1978/79 to 1997/98 IDCR-SOWER surveys. Journal of Cetacean Research and Management 3:143-174

Buckland ST, Bloch D, Cattanach KL, Gunnlaugsson T, Hoydal K, Lens S, Sigurjónsson J (1993a) Distribution and abundance of long-finned pilot whales in the North Atlantic, estimated from NASS-87 and NASS-89 data. In: Donovan GP, Lockyer CH, Martin AR (eds) Biology of Northern Hemisphere Pilot Whales - Reports of the International Whaling Commission (*Special Issue 14*). IWC, Cambridge, UK, p 33-49

Buckland ST, Cattanach KL (1992) Fin whale abundance in the North Atlantic, estimated from Icelandic and Faroes NASS-87 and NASS-89 data. In: Report of the International Whaling Commission, Vol 42. Internatinal Whaling Commission, IWC, p 645-651

Buckland ST, Cattanach KL, Hobbs RC (1993b) Abundance estimates of Pacific white-sided dolphin, northern right whale dolphin, Dall's porpoise and northern fur seal in the North Pacific, 1987-1990. International North Pacific Fisheries Commission Bulletin:387-407

Buckland ST, Cattanach KL, Miyashita T (1992) Minke whale abundance in the northwest Pacific and the Okhotsk Sea, estimated from 1989 and 1990 sighting surveys. Reports of the International Whaling Commission 42:387-392

Burt ML, Borchers DL, Samarra F (2006) Abundance estimates from SCANS-II: Stratified analysis, RUWPA, University of St Andrews, St. Andrews, UK

Calambokidis J, Barlow J (2004) Abundance of blue and humpback whales in the eastern North Pacific estimated by capture-recapture and line-transect methods. Marine Mammal Science 20:63-85

Calambokidis J, Steiger GH, Ellifrit DK, Troutman BL, Bowlby CE (2004) Distribution and abundance of humpback whale (*Megaptera novaeangliae*) and other marine mammals off the northern Washington coast. Fishery bulletin 102:563–580

Cañadas A, Hammond PS (2006) Model-based abundance estimates for bottlenose dolphins off southern Spain: Implications for conservation and management. Journal of Cetacean Research & Management

Carretta JV (2003) Preliminary estimates of harbor porpoise abundance in California from 1997 and 1999 aerial surveys. Report No. Admin. Rept. LJ-03-04, Southwest Fisheries Science Center (SWFSC), National Marine Fisheries Service, (NMFS), National Oceanic and Atmospheric Administration (NOAA), La Jolla, California

Carretta JV, Forney KA (2004) Preliminary estimates of harbour porpoise abundance in California from 1999 and 2002 aerial surveys. Report No. Admin. Rept. LJ-04-01, Southwest Fisheries Science Center (SWFSC), National Marine Fisheries Service, (NMFS), National Oceanic and Atmospheric Administration (NOAA), La Jolla, California

Carretta JV, Taylor BL, Chivers SJ (2001) Abundance and depth distribution of harbor porpoise (Phocoena phocoena) in northern California determined from a 1995 ship survey. Fishery bulletin 99:29-39

Cattanach KL, Sigurjónsson J, Buckland ST, Gunnlaugsson T (1993) Sei whale abundance in the North Atlantic, estimated from NASS-87 and NASS-89 data. In: Reports of the International Whaling Commission, Vol 43, p 315-321

Christensen I, Haug T, Oien N (1992) Seasonal distribution, exploitation and present abundance of stocks of large baleen whales (Mysticeti) and sperm whales, *Physeter macrocephalus*, in Norwegian and adjacent waters. ICES Journal of Marine Science 49:341-355

Clapham PJ, Barlow J, Bessinger M, Cole T, Mattila D, Pace R, Palka D, Robbins J, Seton R (2003) Abundance and demographic parameters of humpback whales from the Gulf of Maine, and stock definition relative to the Scotian Shelf. Journal of Cetacean Research and Management 5:13-22

Danilewicz D, Moreno IB, Ott PH, Tavaresa M, Azevedo A, Secchi ER, Andriolo A (2006) Abundance estimation for a threatened population of franciscana dolphins in southern Brazil (SC/58/SM6) International Whaling Commission, IWC Scientific Committee Meeting. (unpublished), p 11

Davis RW, Evans WE, Wursig B (2000) Cetaceans, Sea Turtles and Seabirds in the Northern Gulf of Mexico: Distribution, Abundance and Habitat Associations. Volume II: Technical Report. Report No. OCS Study MMS 2000-003 - USGS/BRD/CR-1999-0005, Texas A&M University at Galveston and the National Marine Fisheries Service.

Davis RW, Fargion GS (1996) Distribution and Abundance of Marine Mammals in North-Central and Western Gulf of Mexico: Final Report. Volume II: Technical Report. Report No. OCS Study MMS 96-0027, US Department of the Interior, Minerals Management Service

Findlay KP, Meÿer M, Elwen S, Kotze D, Johnson RM, Truter P, Uamusse C, Sitoe S, Wilke C, Kerwath S, Swanson S, Steverees L, van der Westhuizen J (2004) Distribution and abundance of humpback whales, *Megaptera novaeangliae*, off the coast of Mozambique, 2003. Journal of Cetacean Research and Management (Special Issue)

Forcada J, Aguilar A, Hammond PS, Pastor X, Aguilar R (1994) Distribution and numbers of striped dolphins in the western Mediterranean Sea after the 1990 epizootic outbreak. Marine Mammal Science 10:137-150

Forcada J, Gazo M, Aguilar A, Gonzalvo J, Fernandez-Contreras M (2004) Bottlenose dolphin abundance in the NW Mediterranean: Addressing heterogeneity in distribution. Marine Ecology Progress Series 275:275-287

Forcada J, Notarbartolo DSG, Fabbri F (1995) Abundance of fin whales and striped dolphins summering in the Corso-Ligurian Basin. Mammalia 59:127-140

Forney KA (1999) The abundance of California harbor porpoise estimated from 1993–97 aerial line-transect surveys. Report No. LJ-99-02, Southwest Fisheries Science Center (SWFSC), National Marine Fisheries Service, (NMFS), National Oceanic and Atmospheric Administration (NOAA), La Jolla, California

Forney KA, Barlow J (1993) Preliminary winter abundance estimates for cetaceans along the California coast based on a 1991 aerial survey. Reports of the International Whaling Commission:407-415

Forney KA, Barlow J, Carretta JV (1995) The abundance of cetaceans in California waters: Part II. Aerial surveys in winter and spring of 1991 and 1992. US National Marine Fisheries Service Fishery Bulletin 93:15-26

Garrison LP, Swartz S, Martinez A, Burks C, Stamates J (2003) A marine mammal assessment survey of the Southeast US Continental Shelf: February - April 2002. Report No. NMFS-SEFSC-492, U.S. Department of Commerce

Gerrodette T, Forcada J (2002a) Estimates of abundance of northeastern offshore spotted, coastal spotted, and eastern spinner dolphins in the eastern tropical Pacific ocean. Report No. Admin. Rept. LJ-02-06, Southwest Fisheries Science Center (SWFSC), National Marine Fisheries Service, (NMFS), National Oceanic and Atmospheric Administration (NOAA), La Jolla, CA, USA

Gerrodette T, Forcada J (2002b) Estimates of abundance of western/southern spotted, whitebelly spinner, striped and common dolphins, and pilot, sperm and Bryde's whales in the Estern Tropical Pacific Ocean. Report No. Admin. Rept. LJ-02-20, Southwest Fisheries Science Center (SWFSC), National Marine Fisheries Service, (NMFS), National Oceanic and Atmospheric Administration (NOAA), La Jolla, California

Gerrodette T, Watters G, Forcada J (2005) Preliminary estimates of 2003 dolphin abundance in the Eastern Tropical Pacific. Report No. Admin. Rept. LJ-05-05, Southwest Fisheries Science Center (SWFSC), National Marine Fisheries Service, (NMFS), National Oceanic and Atmospheric Administration (NOAA), La Jolla, California

Griffin RB, Griffin NJ (2003) Distribution, habitat partitioning, and abundance of Atlantic spotted dolphins, bottlenose dolphins, and Loggerhead sea turtles on the eastern Gulf of Mexico continental shelf. Gulf of Mexico Science 1:23–34

Gunnlaugsson T, Sigurjónsson J (1990) NASS-87: Estimation of whale abundance based on observations made onboard Icelandic and Faroese survey vessels. Reports of the International Whaling Commission 40:481-483

Hammond PS, Berggren P, Benke H, Borchers DL, Collet A, Heide-Jorgensen MP, Heimlich S, Hiby AR, Leopold MF, Øien N (2002) Abundance of harbour porpoise and other cetaceans in the North Sea and adjacent waters. Journal of Applied Ecology 39:361-376

Hansen LJ, Mullin KD, Roden CL (1995) Estimates of cetacean abundance in the northern Gulf of Mexico from vessel surveys. Report No. Contribution No. MIA-94/95-25, SEFSC, Miami Laboratory, Miami, USA

Harwood LA, Innes S, Norton P, Kingsley MCS (1996) Distribution and abundance of beluga whales in the Mackenzie estuary, southeast Beaufort Sea, and west Amundsen Gulf during late July 1992. Canadian Journal of Fisheries & Aquatic Sciences 53:2262-2273

Heide-Jørgensen MP, Lassen H, Teilmann J, Davis RA (1993) An index of the relative abundance of wintering belugas, *Delphinapterus leucas*, and narwhals, *Monodon monoceros*, off West Greenland. Canadian Journal of Fisheries & Aquatic Sciences 50:2323-2335

Heide-Jørgensen MP, Reeves RR (1996) Evidence of a decline in beluga, *Delphinapterus leucas*, abundance off West Greenland. ICES Journal of Marine Science 53:61-72

Heide-Jørgensen MP, Simon MJ, Laidre KL (2006) Estimates of large whale abundance in Greenland waters from a ship-based survey in 2005 (SC/58/AWMP6) Internation Whaling Commission - Scientific Committee Meeting. (unpublished), St. Kitts, p 28

Hobbs RC, Rugh DJ, DeMaster DP (2000) Abundance of belugas, *Delphinapterus leucas*, in Cook Inlet, Alaska, 1994-2000. Marine Fisheries Review 62:37-45

Kasamatsu F, Joyce GG (1995) Current status of odontocetes in the Antarctic. Antarctic Science 7:365-379

Kingsley MCS (2000) Numbers and distribution of beluga whales, *Delphinapterus leucas*, in James Bay, eastern Hudson Bay, and Ungava Bay in Canada during the summer of 1993. Fishery bulletin 98:736-747

Kingsley MCS, Reeves RR (1998) Aerial surveys of cetaceans in the Gulf of St. Lawrence in 1995 and 1996. Canadian Journal of Zoology 76:1529-1550

Larsen F (1995) Abundance of minke and fin whales off West Greenland, 1993. Reports of the International Whaling Commission 45:365-370

Lowry FL, DeMaster DP, Frost KJ, Perryman W (1999a) Alaska beluga whale committee surveys of beluga whales in the eastern Chukchi Sea, 1992-1995. (SC/51/SM34) International Whaling Commission - Scientific Committee Meeting. (unpublished), Grenada, p 20

Lowry FL, DeMaster DP, Frost KJ, Perryman W (1999b) Alaska beluga whale committee surveys of beluga whales in the eastern Chukchi Sea, 1996-1998. (SC/51/SM33) International Whaling Commission - Scientific Committee Meeting. (unpublished), Grenada, p 20

Lowry LF, Frost KJ (1999) Alaska Beluga Whale Committee surveys of beluga whales in Bristol Bay, Alaska, 1993-1994 (SC/51/SM32) International Whaling Commission - Scientific Committee Meeting. (unpublished), Grenada

MacLeod K (2004) Abundance of Atlantic white-sided dolphin (*Lagenorhynchus acutus*) during summer off northwest Scotland. Journal of Cetacean Research and Management 6:33-40

MacLeod K, Simmonds M, Murray E (2006) Abundance of fin (*Balaenoptera physalus*) and sei whales (*Balaenoptera borealis*) amid oil exploration and development off northwest Scotland. Journal of Cetacean Research and Management 8:247-254

Miyashita T (1986) Abundance of Baird's beaked whales off the Pacific coast of Japan. Reports of the International Whaling Commission 36:383-386

Miyashita T (1993) Abundance of dolphin stocks in the western North Pacific taken by the Japanese drive fishery. Reports of the International Whaling Commission 43:417-437

Miyashita T, Kato H (1993) Population estimate of Baird's beaked whales off the Pacific coast of Japan using sighting data collected by R/V Shunyo Maru in 1991 and 1992. (SC/45/SM6) International Whaling Commission - Scientific Committee Meeting. (unpublished), p 12

Mobley JRJ, Spitz SS, Forney KA, Grotefendt RA, Forestall PH (2000) Distribution and abundance of odontocete species in Hawaiian waters: Preliminary results of 1993-98 aerial surveys. Report No. Administration Report LJ-00-14C, Southwest Fisheries Science Center, National Marine Fisheries Service, La Jolla, California, USA

Moore SE, Waite JM, Friday NA, Honkalehto T (2002) Cetacean distribution and relative abundance on the central-eastern and the southeastern Bering Sea shelf with reference to oceanographic domains. Progress in Oceanography 55:249-261

Moore SE, Waite JM, Mazzuca LL, Hobbs RC (2000) Mysticete whale abundance and observations of prey associations on the central Bering Sea shelf. Journal of Cetacean Research and Management 2:227-234

Mullin KD, Fulling GL (2003) Abundance of cetaceans in the southern U.S. Atlantic ocean during summer 1998. Fishery bulletin 101:603-613

Mullin KD, Fulling GL (2004) Abundance of cetaceans in the oceanic northern Gulf of Mexico, 1996-2001. Marine Mammal Science 20:787–807

O'Cadhla O, Burt ML, Borges AL, Rogan E (2001) Summer distribution and abundance of cetaceans in western Irish waters and the Rockall Trough. SC/51/O15 International Whaling Commission - Scientific Committee Meeting. (unpublished)

Palka D (1995) Abundance estimate of the Gulf of Maine harbor porpoise. In: Bjørge A, Donovan GP (eds) Biology of the phocoenids - Reports of the International Whaling Commission (*Special issue 16*). IWC, Cambridge, UK, p 27-50

Palka D (2006) Summer abundance estimates of cetaceans in US North Atlantic navy operating areas. In: Northeast Fisheries Science Center Reference Document 06-03

Rosenbaum HC, Strindberg S, Ersts PJ (2004) Initial estimates abundance and distribution of humpback whales on their wintering grounds in the coastal waters of Gabon (southeastern Atlantic Ocean , Area B ) based on aerial surveys (SC/56/SH2) International Whaling Commission - Scientific Committee Meeting. unpublished, Sorrento, Italy, p 12

Rugh DJ, Shelden KEW, Sims CL, Mahoney BA, Smith BK, Litzky LK, Hobbs RC (2005) Aerial Surveys of Belugas in Cook Inlet, Alaska, June 2001, 2002, 2003 and 2004. Report No. NMFS-AFSC-149, U.S. Department of Commerce

Scheidat M, Gilles A, Kock K-H, Siebert U (2006) Harbour porpoise (Phocoena phocoena) abundance in German waters (July 2004 and May 2005) (SC/58/SM19) International Whaling Commission - Scientific Committee Meeting. (unpublished), p 11

Schiavini A, Pedraza SN, Crespo EA, Gonzalez R, Dans SL (1999) Abundance of dusky dolphins (*Lagenorhynchus obscurus*) off north and central Patagonia, Argentina, in spring and a comparison with incidental catch in fisheries. Marine Mammal Science 15:828-840

Schweder T, Skaug HJ, Dimakos XK, Langaas M, Oien N (1997) Abundance of northeastern Atlantic minke whales, estimates for 1989 and 1995. In: Donovan GP (ed) Reports of the International Whaling Commission, Vol 47. IWC, p 453-483

Shirakihara K, Shirakihara M, Yamamoto Y (2007) Distribution and abundance of finless porpoise in the Inland Sea of Japan. Marine Biology 150:1025-1032

Siebert U, Gilles A, Lucke K, Ludwig M, Benke H, Kock K-H, Scheidat M (2006) A decade of harbour porpoise occurrence in German waters — Analyses of aerial surveys, incidental sightings and strandings. Journal of Sea Research 56:65-80

Skaug HJ, Øien N, Schweder T, Bøthun G (2004) Current abundance of minke whales in the northeastern Atlantic; variability in time and space. Canadian Journal of Fisheries and Aquatic Sciences 61:870–886

Wade PR, Gerrodette T (1993) Estimates of cetacean abundance and distribution in the Eastern Tropical Pacific. Reports of the International Whaling Commission 43:477-493

Waite JM, Friday NA, Moore SE (2002) Killer whale (*Orcinus orca*) distribution and abundance in the central and southeastern Bering Sea, July 1999 and June 2000. Marine Mammal Science 18:779-786

Zerbini AN, Andriolo A, Da Rocha JM, Simoes-Lopes PC, Siciliano S, Pizzorno JL, Waite JM, DeMaster DP, VanBlaricom GR (2004) Winter distribution and abundance of humpback whales (*Megaptera novaeangliae*) off Northeastern Brazil. Journal of Cetacean Research & Management 6:101-107

Zerbini AN, Waite JM, Durban JW, LeDuc RG, Dahlheim ME, Wade PR (2007) Estimating abundance of killer whales in the nearshore waters of the Gulf of Alaska and Aleutian Islands using line-transect sampling. Marine Biology 150:1033-1045

Zerbini AN, Waite JM, Laake JL, Wade PR (2006) Abundance, trends and distribution of baleen whales off Western Alaska and the central Aleutian Islands. Deep Sea Research Part I: Oceanographic Research Papers 53:1772-1790
